# Chorioamnionitis (ChA) modifies CX3CL1 (fractalkine) production by human amniotic epithelial cells (HAEC) under normoxic and hypoxic conditions

**DOI:** 10.1186/1476-9255-11-12

**Published:** 2014-05-13

**Authors:** Dariusz Szukiewicz, Jan Kochanowski, Tarun Kumar Mittal, Michal Pyzlak, Grzegorz Szewczyk, Krzysztof Cendrowski

**Affiliations:** 1 Department of General & Experimental Pathology, Medical University of Warsaw, ul.Krakowskie Przedmiescie 26/28, Warsaw 00-928, Poland; 2 Department of Neurology, Second Faculty of Medicine, Medical University of Warsaw, Warsaw, Poland; 3 Department of Obstetrics & Gynecology, Second Faculty of Medicine, Medical University of Warsaw, Warsaw, Poland

**Keywords:** Chorioamnionitis, CX3CL1, Fractalkine, Human amniotic epithelial cells, Hypoxia

## Abstract

**Background:**

Chemokine CX3CL1 possesses unique properties, including combined adhesive and chemotactic functions. Human amniotic epithelial cells (HAEC) show expression of CX3CL1 receptor (CX3CR1) and produce CX3CL1 in response to both physiologic and pathologic stimuli. Chorioamnionitis (ChA) is a common complication of pregnancy and labour. ChA is often accompanied by local hypoxia because of the high oxygen consumption at the site of inflammation. We examined comparatively (ChA-complicated vs. normal pregnancy) CX3CR1 expression and the effects of hypoxia, lipopolysaccharide (LPS), and CX3CR1 blockade on CX3CL1 production in HAEC cultured in vitro.

**Methods:**

HAEC have been isolated using trypsinization, and cultured under normoxia (20% O_2_) vs. hypoxia (5% O_2_). According to the experimental design, LPS (1 μg/ml) and neutralizing anti-CX3CR1 antibodies were added at respective time points. Mean CX3CL1 concentration in the supernatant samples were determined by ELISA. Expression of immunostained CX3CR1 was analyzed using quantitative morphometry.

**Results:**

We have found that the mean levels of CX3CL1 and CX3CR1 expression were remarkably (p < 0.05) higher in ChA, compared to normal pregnancy. Significantly increased expression of CX3CR1 was observed in ChA during both normoxia and hypoxia. Hypoxia exposure produced decrease in the mean concentration of CX3CL1 in both groups, however this reduction was stronger in normal pregnancy. In normoxia, LPS-evoked rise in the mean concentration of CX3CL1 was higher (p < 0.05) in normal pregnancy. This response was positively correlated with CX3CR1 expression. Blockade of CX3CR1 canceled the secretory response to LPS in all groups.

**Conclusions:**

ChA-complicated pregnancy up-regulates CX3CR1 in HAEC cultured in vitro with simultaneous increase in CX3CL1 production. Hypoxia-resistant production of CX3CL1 may be responsible for ChA-related complications of pregnancy and labor.

## Background

Chemokines comprise a heterogeneous family of low-molecular mass cytokines (8-15 kDa) [1]. The major roles of chemokines are to act as chemoattractants to guide the migration of leukocytes and to modulate immune responses [2]. Members of the chemokine family are divided into four groups based on the spacing of their first two cysteine residues [3,4]. The chemokine CX3CL1 (known also as fractalkine or neurotactin) was first described in 1997 by Bazan *et al*. and Pan *et al*. [5,6]. Located on human chromosome 16, CX3CL1 is the only CX3C (delta) subfamily member, with three amino acid residues located between the first two cysteine residues in the molecular formula. Unlike other chemokines, CX3CL1 is of non-hemopoietic origin and shows potent chemoattractant properties for natural killer (NK) cells, T cells and monocytes, but not neutrophils [5]. CX3CL1 exists in two forms: as a transmembrane protein with a chemokine domain fixed to a long mucin-like stalk and as a soluble peptide that cleaves from the cell surface [7]. It has been previously shown that the main roles of membrane-bound CX3CL1 include promotion of leukocyte binding and adhesion, as well as activation of target cells. However, the soluble chemokine domain of human CX3CL1 is chemotactic for T cells and monocytes [8]. Its dual role as an adhesive particle and a chemoattractant makes CX3CL1 unique. Most stimuli that influence cell homeostasis potentially induce CX3CL1 secretion [9].

CX3CL1 acts in humans via its receptor, CX3CR1 (previously named V28), which is also known as the fractalkine receptor [10]. This receptor is a member of a large protein family of transmembrane receptors, G protein-coupled receptors (GPCRs). Stimulation of CX3CR1 evokes activation of both CX3CL1-dependent and integrin-dependent migration of cells with augmented adhesion as a result of synergistic reactions [10,11].

It is well understood that physiological phenomena related to normal growth, development and reproduction of living organisms are governed by a complex chemokine network [12-14]. Multiple authors suggest that, to some degree, normal pregnancy is a controlled inflammatory state and that many complications of pregnancy are related to exaggerated inflammatory response (local or systemic) [15,16]. Therefore, a successful pregnancy depends on a balance between anti-inflammatory and pro-inflammatory cytokines [17].

The data regarding the role of CX3CL1 in reproduction are still accumulating [18]. Together with several other cytokines (e.g., CCL7, CCL4, CCL14), CX3CL1 is involved in the processes of implantation, invasion of the trophoblast into the spiral uterine arteries, placental angiogenesis, response to inflammatory and immunologic factors within the utero-placental interface, and induction of labor [19-21].

Human amniotic epithelial cells (HAEC) are released into amniotic fluid by various cytokines, including chemokines and CX3CL1. During the course of pregnancy, the chemokine profile of HAEC may be modified by many factors. Considering that CX3CL1 regulates accumulation of lymphocytes in regions of inflammation, the expression of this chemokine is expected to be up-regulated by inflammatory stimuli [22,23]. However, inflammation is often accompanied by significant local reductions in oxygen availability [24].

Data available from both in vitro and in vivo experiments indicate that hypoxia markedly inhibits production of CX3CL1 [25,26]. This is perplexing because both inflammation and hypoxia produce an increase in the local concentration of tumor necrosis factor alpha (TNF-α), a well-known inducer of CX3CL1 production and CX3CR1 expression [9,27,28].

Chorioamnionitis (ChA), also referred to as inflammation of the fetal membranes (amnion and chorion), is a common complication of pregnancy associated with significant maternal, perinatal and long-term adverse outcomes [29,30]. ChA is caused by a breach in the normal defenses of the uterine interior by bacteria, typically ascending from lower in the vagina. ChA can cause blood infection (bacteremia) in the mother and serious infection in the fetus/newborn. Additionally, ChA may predispose pregnant women to increased uterine contractility and thus a heightened risk of premature labor. The role of CX3CL1 in premature rupture of membranes (PROM) and preterm labor is still under investigation [31].

The aim of this study was to examine and compare ChA-complicated vs. normal-course pregnancy (group I vs. group II, respectively) in respect of the influence of different oxygen tensions (normoxia and hypoxia) on lipopolysaccharide (LPS)-induced CX3CL1 production in HAEC cultured in vitro.

## Materials and methods

### Tissue collection

**Figure 1:**
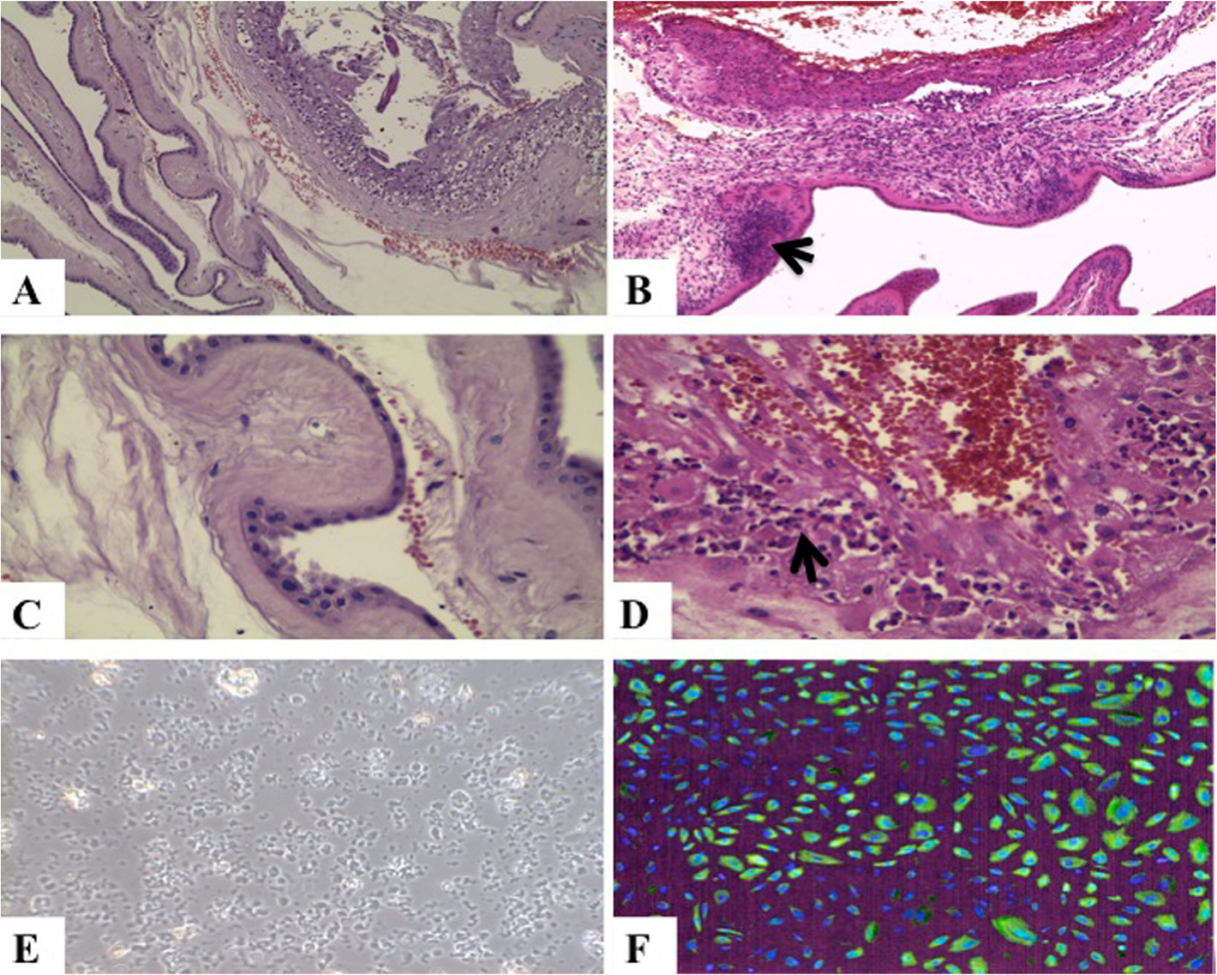
Histologic images: A – normal placental membranes, HE (Hematoxylin & Eosin, the routine staining) x 100; B – chorioamnionitis with perivascular inflammatory infiltrate (arrow head), HE x 100; C – normal amnion, HE x 400; D – chorioamnionitis with perivascular inflammatory infiltrate (arrow head), HE x 400; E – HEAC in culture, phase contrast x 100; F – CX3CR1 immunostained in HAEC culture (image digitally transformed for morphometric purposes), x 200.

This study was conducted in compliance with international and local laws of human experimentation and was officially approved by a local ethics committee of the Medical University of Warsaw, Poland; written consents were obtained from the women for the use of their extraplacental membranes. The study was carried out in accordance with The Code of Ethics of the World Medical Association (Declaration of Helsinki) for experiments involving humans, and the Uniform Requirements for manuscripts submitted to Biomedical Journals have been fulfilled. The membranes in group I (*N* = 14; mean gestational age 269 ± 8 days) were collected after single term pregnancies terminated by cesarean section due to developing symptoms of inflammation without the possibility of the rapid completion of vaginal delivery in patients with rupture of membranes. ChA was diagnosed based on the results of blood tests performed during pregnancy and labor (leukocyte count >15 × 10^9^/L; C-reactive protein concentrations in three consecutive tests >10 mg/L) confirmed by light microscopic evidence of histologic inflammation (infiltrations of inflammatory cells) (Figure 1B,D). The membranes in group II (*N* = 14; singleton pregnancies, mean gestational age 272 ± 7 days) were obtained after elective cesarean sections due to breech presentation of the fetus in primigravida or retinal diseases with contraindications to vaginal delivery (Figure 1A,C).

### Cell culture & determination of CX3CL1 concentration

HAEC were isolated by the method of Okita *et al.* as described elsewhere [32,33]. Briefly, immediately following delivery of the afterbirth (placenta), amnion samples of 6 × 6 cm were manually separated from the chorion, washed with phosphate-buffered saline (PBS) and incubated for 20 min with 5% trypsin (dilution 1:250; BD Difco™) at 37˚C to dispose of adherent cellular debris. After this stage, the trypsin was removed, and HAEC isolated from the basement membrane were subjected to another, extended enzymatic digestion for 90 min at 37°C with 3% trypsin. The cells were then suspended in Ham’s F12/Dulbecco’s modified Eagle medium supplemented with 10% fetal calf serum. HAEC were seeded at a density of 1 × 10^5^ cells/well in 24-well culture plate inserts (BD Falcon, NJ, USA) (Figure 1E). Four HAEC cultures were established from each placenta, resulting in a total of 112 cultures examined in total from both groups (56 per group).

Isolated cells had a polygonal epithelioid appearance with perinuclear inclusions and were almost homogenous (minimum 97% of purity). The vast majority of cells were positive for specific simple epithelial cytokeratin peptide 18 (Abcam, USA) and were negative for specific macrophage/monocyte markers: CD68 (Dako, Denmark) and HLA-DR (Santa Cruz, USA). The cultures were kept in normoxia at 37˚C in a humidified atmosphere (relative humidity of 95%) and reached confluence at approximately day 5 to day 6. At this time, the supernatants were obtained for immunoenzymatic assay (ELISA) of the initial concentration of CX3CL1 in the cultures. The applied method for the determination of CX3CL1 using RayBio® Human Fractalkine ELISA Kit has a very high specificity; to the best of our knowledge, it exceeds other available ELISA tests for detection of CX3CL1 in cell culture media.

**Figure 2:**
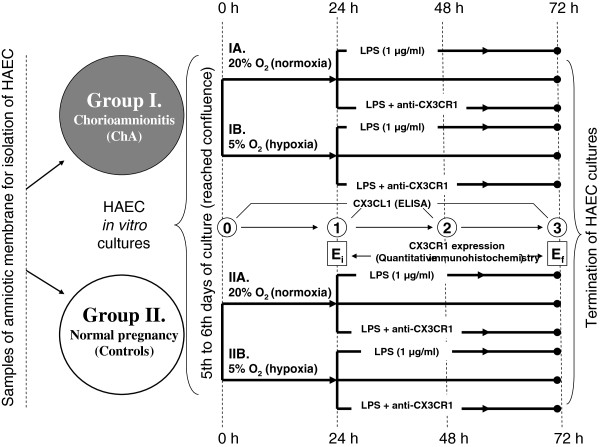
**General scheme of the cultures developed within the study groups and the time frames of the research procedures: 0,1,2,3 – measurements of CX3CL1 concentration in the culture media by ELISA; E**_**i**_**, E**_**f **_**– expressions of CX3CR1 in HEAC cultures (the initial and final, respectively).**

Subsequently, cultured HAEC in both groups were divided into subgroups: normoxic (20% O_2_, 75% N_2_, 5% CO_2_; groups IA and IIA, respectively) and hypoxic (5% O_2_, 90% N_2_, 5% CO_2_; groups IB and IIB, respectively). The levels of CX3CL1 were measured in the subgroups at 24 h, 48 h, and 72 h time points. The influence of lipopolysaccharide (LPS, 1 μg/ml) on the secretory response was also examined. To evaluate the effect of CX3CR1 receptor blockade on CX3CL1 production under hypoxia and normoxia in both the absence and presence of LPS in the culture medium, additional subgroups were formed. To block CX3CR1 in HAEC cultures *in vitro*, rabbit anti-human CX3CR1 "neutralizing" antibodies (dilution 1:300; TP502; Torrey Pines Bipolabs, Inc., NJ, USA) were added to the medium. Respective controls were established within each of the subgroups. A general scheme of the culture development within the groups and time frames of the research procedures is shown in Figure 2.

### Immunohistochemistry and mean expression of CX3CR1

Both the initial (E_i_; normoxia; approximately day 5 to day 6 of culture) and final (E_f_; 72 hours after subsequent continuation of the cell cultures under hypoxia or normoxia) expression levels of fractalkine receptor CX3CR1 were compared between the study groups. To perform this task, some of the cultures within the study groups (*N* = 24 per group) were formalin fixed and paraffin embedded at the above-mentioned time points. CX3CR1 was visualized using a standard immunohistochemical procedure. Rabbit polyclonal antibody IgG to CX3CR1 (ab8020; Abcam Inc., USA; concentration of 10 μg/ml) was used as the primary antibody, and goat anti-rabbit IgG was used as the biotinylated secondary antibody (ab64256; Abcam; 0.5% v/v). The visualization of primary anti-receptor antibodies was conducted using a StreptABComplex/HRP Duet (Dako Cytomation, Glostrup, Denmark) following the procedure recommended by the manufacturer, with 3,3’-diaminobenzidine serving as a chromogen. Negative controls for the immunostaining were set up by replacement of the polyclonal primary antibody with normal rabbit preimmune IgG diluted with phosphate buffered saline, containing 3% bovine serum albumin at the same protein concentration as that used for the primary antibody.

Quantitative immunohistochemistry using morphometric software (Quantimet 500C+, Leica, UK) was applied for CX3CR1 receptor identification in paraffin 5 μm sections of the HAEC cultures under light microscopy (Figure 1F). All morphometric procedures were carried out twice by two independent observers, and the average results were recorded. The intensity of immunostaining was evaluated using mean color saturation parameters and thresholds in grey-level histograms. Expression of CX3CR1 corresponded to the total immunostained calibrated area of examined sections, where color saturation comprises segmentation-separation criteria for objects. Each single analyzed image area was 138 692 μm^2^ (magnification × 200). In each group, 72 visual fields were analyzed (36 visual fields per each time point marked E_i_ and E_f_ as previously stated). To assure optimal accuracy of the measurements, the following factors were controlled or monitored: illumination, power supply, warming up, shading correction, averaging of image intake, hue, luminance, relation of illumination to quantification of area percentage of positively staining structures. More detailed descriptions of these morphometric procedures have been given elsewhere [34,35]. Morphometric results with 90% confidence intervals were reported as the mean percentage values ± SEM.

### Determination of TNF-α concentration

The measurement of the TNF-α levels in the culture supernatants was performed for all studied HAEC groups by ELISA. A commercially available kit was used (ELH-TNFalpha-001, RayBio Human TNF-alpha ELISA Kit, RayBiotech, Inc., USA) according to the manufacturer’s instructions. The minimum detectable dose of TNF-α was established as less than 30 pg/ml.

### Statistical analysis

Mann-Whitney’s U-test was applied. The results are expressed as the means ± SEM or the mean percentage values ± SEM. Differences between groups I and II (ChA-complicated vs. normal-course pregnancy, respectively) were deemed statistically significant if p < 0.05.

## Results

### CX3CL1 concentration

**Figure 3:**
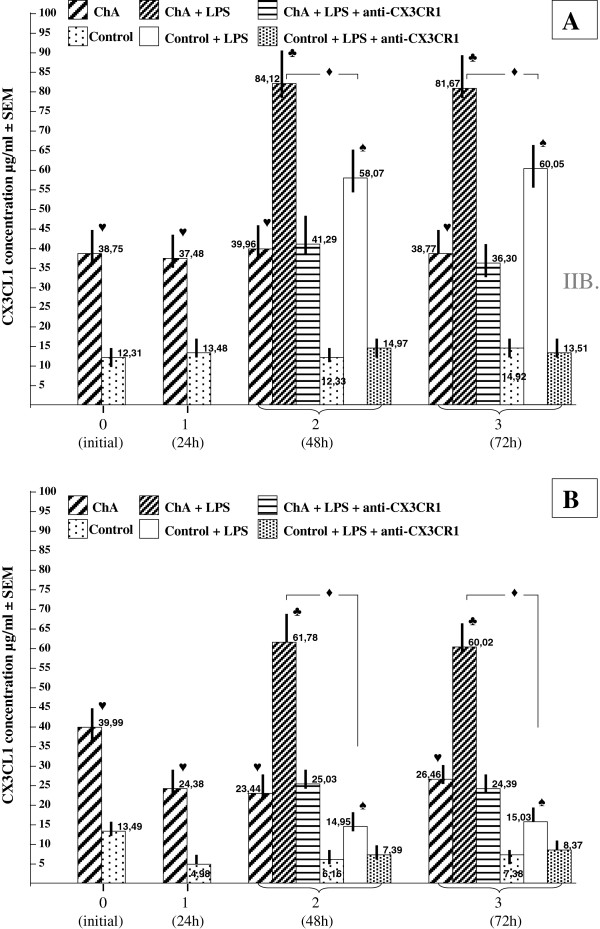
**Mean concentration of CX3CL1 in the culture media in normoxic (A) and hypoxic (B) cultures of HAEC: chorioamnionitis (ChA) versus normal pregnancy (Control).** Symbols ♥, ♦, ♣, ♠ indicate p < 0.05.

Chemokine CX3CL1 was present in of the culture media of all collected specimens. The immunoenzymatic assay procedure (ELISA) used in this study revealed significant (p < 0.05) differences in the mean CX3CL1 concentration between the two main groups as well as between the subgroups, including altered secretory responses of HAEC to LPS challenge (see Figure 3A and B). Immediately after in vitro establishment of the HAEC cultures (5-6 days following enzymatic digestion of the amnion samples), the mean levels of CX3CL1 were significantly (p < 0.05) higher in ChA compared to normal pregnancy (controls) specimens and amounted to 38.75 ± 7.48 and 39.99 ± 7.11 vs. 12.31 ± 3.28 and 13.49 ± 2.65 (μg/ml; subgroups IA, IB vs. IIA, IIB, respectively).

In normoxia, these initial differences in CX3CL1 concentration were observed during the next 3 consecutive assays: after 24, 48, and 72 hours (Figure 3A).

Hypoxia exposure for 24 hours resulted in a decrease in the mean concentration of CX3CL1 in both groups. However, this reduction was stronger in the control group (normal pregnancy), amounting to a 63% vs. 39% drop in the CX3CL1 concentration measured in ChA (Figure 3B).

In repeated measurements performed every 24 hours (48 h and 72 h after establishment of the cultures, respectively), these significant changes in CX3CL1 levels in culture media persisted almost unchanged.

Supplementation of the culture media with LPS resulted in an increased synthesis of CX3CL1 in all studied subgroups, as measured at the 48 h and 72 h time points. The characteristics of this secretory response to LPS have been shown to have strong linkage to the oxygen availability.

In normoxia, LPS-evoked rise in the mean concentration of CX3CL1 was significantly (p < 0.05) higher in the control group (normal pregnancy) compared to the ChA group (2.2-fold vs. 4.3-fold increase, respectively; Figure 3A). This evolving pattern of secretory response to LPS was observed in the control group specimens during HAEC culture under low oxygen tension (Figure 3B). The mean increase in CX3CL1 concentration was reduced almost 2-fold compared to normoxia (2.2-fold vs. 4.3-fold, control group under hypoxia and normoxia, respectively; p < 0.05), while relative magnitude of the mean increase in CX3CL1 remained unchanged in ChA (2.2-fold vs. 2.2-fold, ChA under hypoxia and normoxia, respectively).

Blockade of the CX3CL1-CX3CR1 interaction through administration of “neutralizing” antibodies (rabbit anti-human CX3CR1) resulted in complete inhibition of the secretory response to LPS in all studied groups/subgroups. The differences in the mean CX3CL1 concentrations between LPS-free cultures and “LPS + anti-CX3CR1” cultures were not statistically significant (Figure 3A,B).

### Expression of CX3CR1

**Figure 4:**
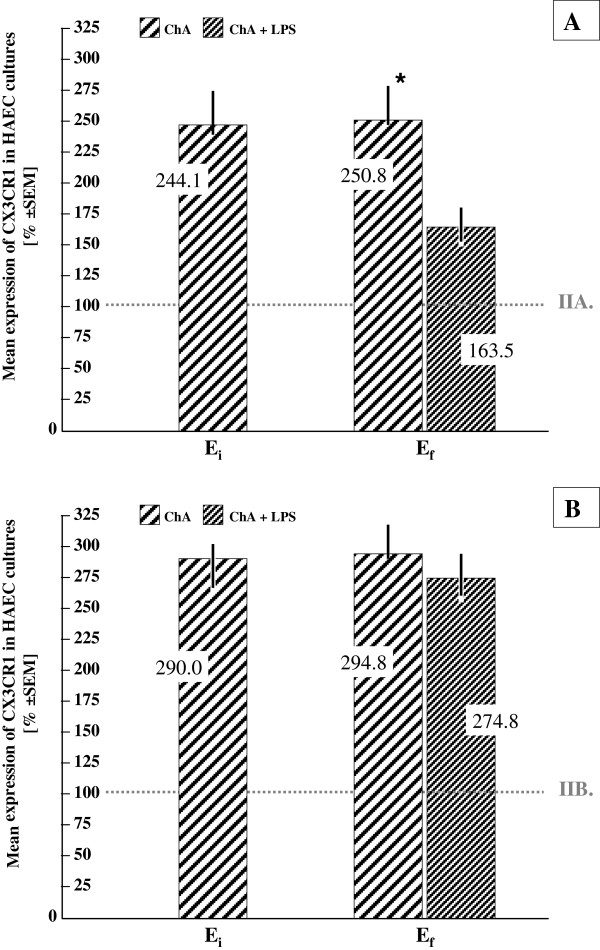
**Mean initial (E**_**i**_**) and final (E**_**f**_**) expressions of CX3CR1 in HAEC cultured in vitro in normoxia (A) and hypoxia (B): chorioamnionitis (ChA) versus normal pregnancy.** Mean values for normal pregnancy (0.077 ± 0.0031 and 0.043 ± 0.0024 for the subgroup IIA, and 0.029 ± 0.0018 and 0.019 ± 0.0013 for the subgroup IIB; abstract numbers ± SEM with or without LPS, respectively) were taken as 100%. * p < 0.05.

The mean percentage values of the initial (E_i_) and the final (E_f_) CX3CR1 receptor expressions were significantly (p < 0.05) higher in ChA under both normoxic and hypoxic conditions, compared to the controls (normal pregnancy) (Figure 4A,B).

Administration of LPS in normoxia produced significant (p < 0.05) differences in the percentage proportions of CX3CR1 expression between the ChA group and the controls. The mean expression of CX3CR1 amounted to 163.5 ± 26.1 and 250.8 ± 31.3% ± SEM (ChA + LPS vs. LPS-free ChA, respectively) of the values in the control group (taken as 100%) (Figure 4A).

Under hypoxia, the mean expression of CX3CR1 following LPS administration in ChA remained approximately proportionally unchanged compared with the percentage values obtained from the LPS-free ChA group (274.8 ± 32.3 and 294.8 ± 28.2% ± SEM, respectively) vs. the mean CX3CR1 expression in the control group taken as 100% (p > 0.05) (see Figure 4B). In other words, the presence of LPS in hypoxic HAEC cultures did not significantly change the differences in CX3CR1 expression observed between the study groups.

### Normoxia vs. hypoxia

Summarizing the data about CX3CL1 concentration and CX3CR1 expression obtained in both groups, we observed the general tendencies in normoxia and hypoxia.

In normoxic conditions the mean CX3CL1 concentration was significantly increased in ChA, compared to the controls (see Figure 3A).

Hypoxia exposure produced significant decrease in CX3CL1 concentration in both goups. The percentage value of this decrease was higher in the control group (normal pregnancy) (see Figure 3B).

The observed secretory response to LPS have also been shown to be oxygen dependent. In normoxia, the mean percentage increase in the mean CX3CL1 concentration was significantly higher in the normal pregnancy compared to the ChA (Figure 3A), while hypoxic conditions produced a two-fold reduction of this secretory response in group II (normal pregnancy) with almost not affected relative magnitude of the mean increase in CX3CL1 in ChA (group I) (Figure 3B).

The mean percentage values of CX3CR1 receptor expressions were significantly higher in ChA, regardless of whether HAEC were grown in normoxia or hypoxia (see Figure 4). Adminitration of LPS in normoxia produced significant reduction in the mean percentage CX3CR1 expression in ChA (Figure 4A), while under hypoxic conditions the influence of LPS on the CX3CR1 expression was not significant (Figure 4B).

### TNF-α concentration

We found that the TNF-α concentration in the culture supernatants was below the limits of detection of the ELISA kit used in our experimental procedure and was not affected by hypoxia and/or administration of LPS (data not shown).

## Discussion

Humoral regulation of the compensatory-adaptive capabilities of the utero-feto-placental unit to meet the changing conditions related to the advancement of normal or complicated pregnancy and the onset of labor is not clearly understood [36,37]. The effects of hormones and autacoids are studied using both in vivo and in vitro models, including isolated HAEC cultures [38,39]. The main reproductive events, including ovulation, implantation and parturition, are all related to inflammatory-like responses [40,41]. In contrast, pathologically augmented inflammatory background (systemic or localized) has been reported in patients with recurrent pregnancy loss or habitual abortion, preterm labor and preterm rupture of the membranes (PROM), as well as diabetic pregnancy and pregnancy-induced hypertension (preeclampsia, eclampsia) [42-45]. The role of HAEC in this inflammatory response modulation and possible interactions with the intrauterine cytokine network during pregnancy remain under investigation [46,47]. To the best of our knowledge, this is the first paper to report the importance of CX3CR1 expression and its functional status in the maintenance of CX3CL1 production by HAEC under hypoxic conditions.

It is crucial to note that similar to all experimental in vitro models, our approach has limitations. Compared to in vivo conditions, isolated HAEC are separated from the influence of natural environmental characteristics, including tight interaction of cytokines that form a cytokine network [12,48]. The increased initial concentration of CX3CL1 in HAEC culture media obtained after ChA-complicated pregnancy seems to reflect the previous stimulatory influence of infective and inflammatory factors on the production and release of this chemokine [31]. It is likely that infection-induced production of inflammatory cytokines, such as TNF-α, IL-1, and IFN-γ, may stimulate CX3CR1 production in HAEC [49,50]. Induction of members of the CX3C chemokine class, such as CX3CL1 in ChA, was relatively stable in normoxia, as the differences between the groups (ChA vs. normal pregnancy) persisted approximately unchanged for the subsequent 3 days. The differences in CX3CL1 concentration between the groups observed under hypoxic conditions may suggest that specific “preconditioning” in the inflammatory environment in vivo was related to the acquired resistance of ChA cells to decreased oxygenation in culture. It is well documented that infection-induced inflammation may lead to local and systemic ischemia as a defense mechanism against pathogens [51,52]. Both inflammation and hypoxia may trigger TNF-α release in vivo, which has been considered a potent up-regulator of the CX3CL1/CX3CR1 signaling pathway [53,54]. Thus, even if CX3CL1/CX3CR1 genes are under negative regulation (suppression) by hypoxia, CX3CL1 persisted at significantly higher levels in the culture supernatant in group I compared to HAEC derived from a normal pregnancy (group II). Higher levels of CX3CL1 were accompanied by an approximately 2.5-fold to nearly 3-fold increase in expression of CX3CR1 in the ChA group (normoxia and hypoxia, respectively; Figure 4).

Although the measured CX3CL1 levels were higher in ChA, administration of LPS in normoxic environments revealed a significantly higher potential for CX3CL1 production within normal HAEC (control group), where the relative increase in CX3CL1 concentration was approximately 2-fold higher when compared to ChA-derived cell culture (group I; Figure 3A). Interestingly, over a similar time period, the percentage differences between the groups (ChA vs. control) in the mean CX3CR1 expression were smallest, suggesting a significant up-regulation of CX3CR1 in the control group (normal HAEC; Figure 4A). In hypoxic environments, the potential of normal HAEC for CX3CL1 production disappeared, with an accompanying return of the percentage differences in the mean CX3CR1 expression to the values previously observed without LPS (Figure 4B). The effect of neutralizing antibodies on CX3CL1 production showed a superior importance of CX3CR1 functional status over CX3CR1 expression both in normoxic and hypoxic environments. Accordingly, it is indicated that co-expression of CX3CL1 and CX3CR1 in HAEC leads to an autocrine effect of CX3CL1-induced CX3CL1 production. This mechanism of autoregulation via CX3CR1, mediated by heterodimeric G-proteins, phosphoinositide (PI) 3-kinase, phosphoinositide-dependent kinase-1 (PDK1), serine/threonine protein kinase (Akt), IқB kinase (IKK) and nuclear factor қB (NF-қB) has been proposed for other cell types [55-57].

Considering that TNF-α in all culture supernatants demonstrated no measurable activity (concentrations below the detectable limit), both LPS-induced CX3CL1 production and augmented CX3CR1 expression seem to occur in a TNF-α-independent manner. Involvement of interleukins and interferons other than TNF-α in LPS-mediated signaling pathways in HAEC cultured in vitro is expected [23,58]. However, in our study, we did not measure the levels of these cytokines in culture media.

More precise disclosure of the observed positive correlation between CX3CL1 levels and CX3CR1 expression is still needed. It is expected that CX3CR1 polymorphisms are of biological significance. Substitution of valine by isoleucine (V249I) and threonine by methionine (T280M) in the coding region of CX3CR1 gene has been previously reported [59]. The presence of these two single-nucleotide polymorphisms and the two consequentially modified alleles may produce opposite effects on cell adhesion. Studies using endothelial cells revealed that the 280M allele decreased cell adhesion, whereas the 249I allele was associated with augmented or nearly unchanged cell adhesion [60,61].

## Conclusions

In the present study, we demonstrated that ChA during pregnancy up-regulates CX3CR1 expression on HAEC in vitro with simultaneous increase in CX3CL1 production. This response persists in HAEC culture both in normoxia and hypoxia and may be augmented by LPS acting through one or more TNF-α-independent mechanisms. Further research may be helpful in translating the in vitro results into in vivo applications, including the possibility of application into clinical obstetric practical treatment with CX3CR1 antagonists in ChA cases.

## Abbreviations

Act: Serine/threonine protein kinase; ChA: Chorioamnionitis; CX3CR1: CX3CL1(fractalkine) receptor; Ei: E_f_, Initial and final expression of CX3CR1, respectively; ELISA: Immunoenzymatic assay (enzyme-linked immunosorbent assay); GPRSs: G protein-coupled receptors; HAEC: Human amniotic epithelial cells; IFN-γ: Interferon gamma; IKK: IқB kinase complex; IL-1: Interleukin 1; LPS: Lipopolysaccharide; NF-қB: Nuclear factor қB; NK cells: Natural killer cells; PBS: Phosphate-buffered saline; PDK1: Phosphoinositide-dependent kinase-1; PI3-kinase: Phosphoinoisitide 3-kinase; PROM: Premature rupture of membranes; TNF-α: Tumor necrosis factor alpha.

## Competing interests

The authors declare that they have no competing interests.

## Authors’ contributions

DS: the study design, cell culture techniques control, quantitative morphometry, statistical analysis and writing of the manuscript; JK: participation in the study design, data analysis and interpretation, quality assurance and monitoring the course of the study; TKM: the tissue collection, the initial stages of HAEC isolation procedure; MP: all data acquisition, HAEC isolation and cell cultures, immunohistochemistry; GS: HAEC cultures, ELISA; KC: the tissue collection process supervising, statistical analysis, critical review of the manuscript. All authors read and approved the final manuscript.
